# Orally Administered Melatonin Prevents Lipopolysaccharide-Induced Neural Tube Defects in Mice

**DOI:** 10.1371/journal.pone.0113763

**Published:** 2014-11-24

**Authors:** Lin Fu, Zhen Yu, Yuan-Hua Chen, Mi-Zhen Xia, Hua Wang, Cheng Zhang, Fang-Biao Tao, De-Xiang Xu

**Affiliations:** 1 Department of Toxicology, Anhui Medical University, Hefei, China; 2 Anhui Provincial Key Laboratory of Population Health & Aristogenics, Anhui Medical University, Hefei, China; 3 School of Life Science, Anhui Medical University, Hefei, China; CHU Sainte Justine and University of Montreal, Canada

## Abstract

Lipopolysaccharide (LPS) has been associated with adverse pregnant outcomes, including fetal demise, intra-uterine growth restriction (IUGR), neural tube defects (NTDs) and preterm delivery in rodent animals. Previous studies demonstrated that melatonin protected against LPS-induced fetal demise, IUGR and preterm delivery. The aim of the present study was to investigate the effects of melatonin on LPS-induced NTDs. All pregnant mice except controls were intraperitoneally injected with LPS (25 µg/kg) daily from gestational day (GD)8 to GD12. Some pregnant mice were orally administered with melatonin (MT, 50 mg/kg) before each LPS injection. A five-day LPS injection resulted in 27.5% of fetuses with anencephaly, exencephaly or encephalomeningocele. Additional experiment showed that maternal LPS exposure significantly down-regulated placental *proton-coupled folate transporter* (*pcft*) and disturbed folate transport from maternal circulation through the placentas into the fetus. Interestingly, melatonin significantly attenuated LPS-induced down-regulation of placental *pcft*. Moreover, melatonin markedly improved the transport of folate from maternal circulation through the placentas into the fetus. Correspondingly, orally administered melatonin reduced the incidence of LPS-induced anencephaly, exencephaly or encephalomeningocele. Taken together, these results suggest that orally administered melatonin prevents LPS-induced NTDs through alleviating LPS-induced disturbance of folate transport from maternal circulation through the placenta into the fetus.

## Introduction

Lipopolysaccharide (LPS) is a toxic component of cell walls in gram-negative bacteria and is widely present in the digestive tracts of humans and animals [1]. Humans are constantly exposed to a low concentration of LPS through infection. Gastrointestinal inflammatory diseases and excess alcohol intake are known to increase permeability of LPS from gastrointestinal tract into blood [2]. LPS has been associated with adverse pregnant outcomes in rodent animals. According to an earlier report, mice exposed to LPS at early gestational stage caused embryonic resorption [3]. We and others showed that mice exposed to LPS at late gestational stage resulted in fetal demise, intra-uterine growth restriction (IUGR), skeletal development retardation, and preterm delivery [4]–[8]. Recently, we found that mice exposed to LPS at middle gestational stage caused neural tube defects (NTDs) [9], [10].

The mechanism of LPS-induced adverse pregnant outcomes has been extensively studied. Several reports have demonstrated that production of excess reactive oxygen species partially contributes to LPS-induced fetal demise, IUGR and NTDs [8], [10]. Moreover, inflammatory cytokines including tumor necrosis factor alpha (TNF-α) are involved in LPS-induced preterm delivery, fetal demise and IUGR [11], [12]. On the other hand, melatonin, the major secretory product of the pineal gland, has antioxidant and anti-inflammatory effects. As a potent antioxidant, melatonin and its metabolites directly scavenge a variety of free radicals [13]–[18]. As an anti-inflammatory agent, melatonin inhibits LPS-evoked inflammatory cytokines and chemokines [19]–[23]. An earlier report from our laboratory showed that melatonin protected mice from LPS-induced fetal demise and IUGR through its anti-inflammatory effect [24]. According to a recent study, melatonin prevents LPS-induced preterm delivery [25].

In the present study, we investigated the effects of melatonin on LPS-induced NTDs. We showed that orally administered melatonin protected mice from LPS-induced NTDs and skeletal development retardation. We demonstrate for the first time that melatonin improves folate transport from maternal circulation into the fetus through attenuating LPS-induced down-regulation of placental folate transporters.

## Materials and Methods

### Chemicals and reagents

Lipopolysaccharide (*Escherichia coli* LPS, serotype 0127:B8) and melatonin were purchased from Sigma Chemical Co. (St. Louis, MO, USA). TRI reagent was from Molecular Research Center, Inc (Cincinnati, Ohio, USA). RNase-free DNase was from Promega Corporation (Madison, WI, USA). All the other reagents were from Sigma or as indicated in the specified methods.

### Animals and treatments

The ICR mice (8∼10 week-old; male mice: 28∼30 g; female mice: 24∼26 g) were purchased from Beijing Vital River whose foundation colonies were all introduced from Charles River Laboratories, Inc. The animals were allowed free access to food and water at all times and were maintained on a 12-h light/dark cycle in a controlled temperature (20–25°C) and humidity (50±5%) environment for a period of 1 week before use. For mating purposes, four females were housed overnight with two males starting at 9:00 P.M. Females were checked by 7:00 A.M. the next morning, and the presence of a vaginal plug was designated as gestational day (GD) 0. To investigate the effects of orally administered melatonin on LPS-induced NTDs, pregnant mice were divided into four groups randomly. In LPS group, fifteen pregnant mice were intraperitoneally (i.p.) injected with LPS (25 µg/kg) daily at 9:00 AM from GD8 to GD12. In LPS+melatonin group, sixteen pregnant mice were orally administered with melatonin (50 mg/kg) by gavage 1 h before each LPS injection. In control group, fifteen pregnant mice were i.p. injected with normal saline (NS) daily at 9:00 AM from GD8 to GD12. In melatonin alone group, sixteen pregnant mice were orally administered melatonin (50 mg/kg) by gavage 1 h before each NS injection. The doses of melatonin used in the present study referred to others [26]. All dams were sacrificed at 9:00 AM on GD18 and gravid uterine weights were recorded. For each litter, the numbers of live fetuses, dead fetuses and resorption sites were counted. Live fetuses were weighed and examined for external morphological malformations. All fetuses were then stored in ethanol a minimum of two weeks for subsequent skeletal evaluation. To investigate the effects of orally administered melatonin on folate contents in maternal sera and mouse embryos, forty-eight pregnant mice were divided into four groups randomly. In LPS group, pregnant mice were i.p. injected with LPS (25 µg/kg) daily at 9:00 AM from GD8 to GD12. In LPS+melatonin group, pregnant mice were orally administered with melatonin (50 mg/kg) by gavage 1 h before each LPS injection. In control group, pregnant mice were i.p. injected with NS daily at 9:00 AM from GD8 to GD12. In melatonin alone group, pregnant mice were orally administered melatonin (50 mg/kg) by gavage 1 h before each NS injection. Pregnant mice were sacrificed 12 h after the last LPS injection. Maternal sera and embryos were collected for measurements of folate contents. To investigate the effects of melatonin on the expression of placental folate transportors, twenty-four pregnant mice were divided into four groups randomly. In LPS group, pregnant mice were i.p. injected with a single dose of LPS (25 µg/kg) at 9:00 AM on GD10. In LPS+melatonin group, pregnant mice were pretreated with melatonin (50 mg/kg) by gavage 1 h before LPS injection. In control group, pregnant mice were i.p. injected with NS at 9:00 AM on GD10. In melatonin alone group, pregnant mice were administered with melatonin (50 mg/kg) by gavage 1 h before NS injection. All pregnant mice were sacrificed 12 h after LPS injection. Placentas were collected for real-time RT-PCR. To investigate the effects of orally administered melatonin on LPS-induced inflammatory cytokines, twenty-four pregnant mice were divided into four groups randomly. In LPS group, pregnant mice were i.p. injected with a single dose of LPS (25 µg/kg) at 9:00 AM on GD10. In LPS+melatonin group, pregnant mice were orally administered with melatonin (50 mg/kg) by gavage 1 h before LPS injection. In control group, pregnant mice were i.p. injected with NS at 9:00 AM on GD10. In melatonin alone group, pregnant mice were orally administered melatonin (50 mg/kg) by gavage 1 h before NS injection. Pregnant mice were sacrificed 2 h after LPS injection. Placentas were collected for real-time RT-PCR. All animals were euthanized with carbon dioxide and cervical dislocation. This study was approved by the Association of Laboratory Animal Sciences and the Center for Laboratory Animal Sciences at Anhui Medical University (Permit Number: 13-0012). All procedures on animals followed the guidelines for humane treatment set by the Association of Laboratory Animal Sciences and the Center for Laboratory Animal Sciences at Anhui Medical University.

### Skeletal examination and evaluation

The fetuses stored in ethanol were cleared of skin, viscera and adipose tissue. Fetuses were then incubated in acetone overnight and subsequently macerated and stained with alizarin red S for 2 d. After an overnight incubation in 70% ethanol/glycerol/benzyl alcohol, the fetuses were stored in glycerol until examination. Skeletal evaluation included determination of the degree of ossification of the phalanges, metacarpals, vertebrae, sternatrae and skull. The size of the anterior fontanel and ossification of the supraoccipital was scored.

### Measurement of folate

Maternal sera and embryos on GD12 were collected for the measurement of folate concentrations. Embryos were homogenized in 3 ml saline. The homogenates were then centrifuged at 3500 rpm for 10 minutes and stored at −80°C until the measurement of folate. The levels of folate were measured by electrochemiluminescence immunoassay, using a kit from Roche (Roche Diagnostics GmbH, Mannheim, Germany) according to the manufacturer's instructions.

### Isolation of total RNA and real-time RT-PCR

Total RNA in mouse placenta was extracted using TRI reagent. RNase-free DNase-treated total RNA (1.0 µg) was reverse-transcribed with AMV (Pregmega). Real-time RT-PCR was performed with a LightCycler 480 SYBR Green I kit (Roche Diagnostics GmbH) using gene-specific primers as listed in Table 1. The amplification reactions were carried out on a LightCycler 480 Instrument (Roche Diagnostics GmbH) with an initial hold step (95°C for 5 minutes) and 50 cycles of a three-step PCR (95°C for 15 seconds, 60°C for 15 seconds, 72°C for 30 seconds).

**Table 1:** Primers for real-time RT-PCR.

Gene	Sequence	Length
*gapdh*	Forward: 5′-ACCCCAGCAAGGACACTGAGCAAG-3′	109
	Reverse: 5′- GGCCCCTCCTGTTATTATGGGGGT-3′	
*tnf-*α	Forward: 5′- CCCTCCTGGCCAACGGCATG-3′	109
	Reverse: 5′-TCGGGGCAGCCTTGTCCCTT-3′	
*il-6*	Forward: 5′-AGACAAAGCCAGAGTCCTTCAGAGA-3′	146
	Reverse: 5′- GCCACTCCTTCTGTGACTCCAGC-3′	
*mcp-1*	Forward: 5′-GGCTGGAGAGCTACAAGAGG-3′	93
	Reverse: 5′- GGTCAGCACAGACCTCTCTC-3′	
*mip-2*	Forward: 5′- TTGCCTTGACCCTGAAGCCCCC -3′	175
	Reverse: 5′- GGCACATCAGGTACGATCCAGGC -3	
*pcft*	Forward: 5′- CTACCCTACCTCACCAGCCT-3′	119
	Reverse: 5′- GCAAACGCAAAGACCACCAT-3′	
*fr*α	Forward: 5′-GTGGAGACAAAGAAGCCCGA-3′	104
	Reverse: 5′-CTCCACTCCCTGCTTAGGGT-3′	

### Statistical analysis

The litter was considered the unit for statistical comparison among different groups. Fetal mortality was calculated per litter and then averaged per group. The incidence of fetal malformation with NTDs were calculated in litter and then averaged in each group. For fetal weight, crown-rump length, and skeletal evaluation, the means were calculated per litter and then averaged per group. Quantified data were expressed as means ± S.E.M. at each point. *P*<0.05 was considered statistically significant. ANOVA and the Student-Newmann-Keuls post hoc test were used to determine differences between the treated animals and the control and statistical significance.

## Results

### Melatonin prevents LPS-induced NTDs

**Figure 1:**
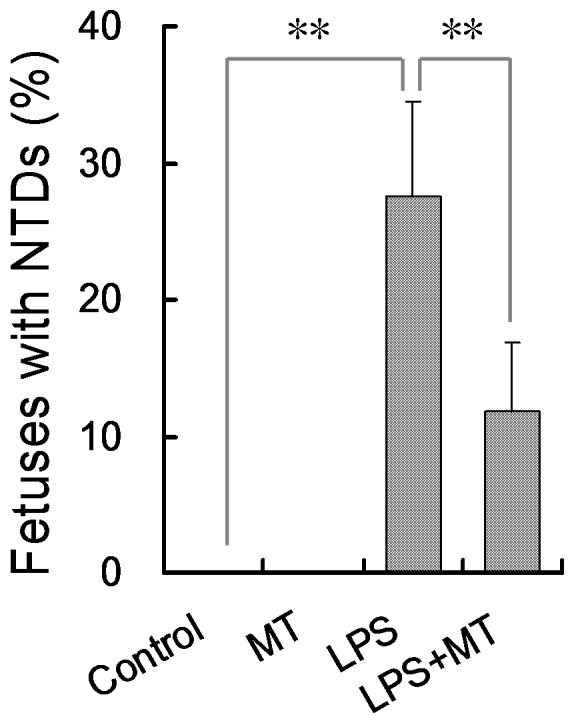
Orally administered melatonin prevents LPS-induced NTDs. In LPS group (n = 15), pregnant mice were i.p. injected with LPS (25 µg/kg) daily from GD8 to GD12. In LPS+melatonin (MT) group (n = 16), pregnant mice were orally administered with melatonin (50 mg/kg) 1 h before each LPS injection. In control group (n = 16), pregnant mice were i.p. injected with NS daily from GD8 to GD12. In melatonin alone group (n = 15), pregnant mice were orally administered with melatonin (50 mg/kg) 1 h before each NS injection. All pregnant mice were sacrificed on GD18. Live fetuses were examined for NTDs. Fetal malformations were calculated in litter and then averaged in each group. Data were presented as means ± S.E.M. ^**^ *P*<0.01, ^*^ *P*<0.05.

Orally administered melatonin had no effect on fodder consumption and weight gain of the pregnant mice (data not shown). No dams died throughout the pregnancy and no abortion was observed. No preterm delivery was observed in dams that completed the pregnancy. The number of litters, live fetuses per litter and dead fetuses per litter were analyzed. There was no significant difference in the number of resorptions per litter and live fetuses per litter among different groups. As shown in Table 2, the number of dead fetuses per litter was markedly increased in LPS-treated mice. Of interest, orally administered melatonin had little effect on LPS-induced fetal demise. The effects of melatonin on LPS-induced NTDs were analyzed. As shown in Table 2, a five-day LPS injection resulted in 66.67% (10/15) of litters with NTDs. Anencephaly, exencephaly and encephalomeningocele are three of the most common NTDs. Among dams injected with LPS, 27.50% of fetuses per litter were with either anencephaly or exencephaly or encephalomeningocele (Figure 1). Of interest, the rate of litters with NTDs was reduced to 43.75% (7/16) when pregnant mice were orally administered with melatonin before LPS injection (Table 2). The incidence of fetuses with NTDs dropped to 13.75% in dams orally administered with melatonin before LPS injection (Figure 1).

**Table 2:** Fetal outcomes among different groups.

	Control	MT	LPS	LPS+MT
Number of litters(n)	16	15	15	16
Number of litters with NTDs [n(%)]	0	0	10 (66.67)	7 (43.75)
Resorptions per litter (n)	1.0±0.4	0.9±0.3	1.3±0.4	1.2±0.4
Dead fetuses per litter(n)	0.2±0.1	0.2±0.2	3.0±1.0**	2.1±0.7
Numbers of live fetuses(n)	197	192	145	161
Live fetuses per litter (n)	12.3±0.7	12.8±0.6	9.7±1.0*	10.1±1.1
Fetal weight(g)	1.40±0.02	1.35±0.02	1.32±0.04	1.30±0.05
Crown-rump length (cm)	2.41±0.02	2.35±0.02	2.36±0.03	2.34±0.03
Placenta weight (g)	0.107±0.003	0.103±0.003	0.104±0.004	0.111±0.005

All data were expressed as means ± SEM.

*P<0.05,

**P<0.01 as compared with the control.

### Melatonin alleviates LPS-induced skeletal malformation

**Figure 2:**
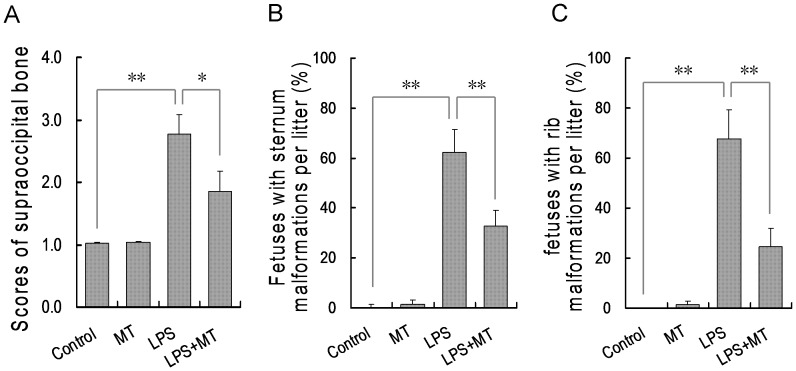
Orally administered melatonin protects against LPS-induced skeletal malformations. n LPS group (n = 15), pregnant mice were i.p. injected with LPS (25 µg/kg) daily from GD8 to GD12. In LPS+melatonin (MT) group (n = 16), pregnant mice were orally administered with melatonin (50 mg/kg) 1 h before each LPS injection. In control group (n = 16), pregnant mice were i.p. injected with NS daily from GD8 to GD12. In melatonin alone group (n = 15), pregnant mice were orally administered with melatonin (50 mg/kg) 1 h before each NS injection. All pregnant mice were sacrificed on GD 18. Live fetuses were examined for skeletal malformations. (A) Supraoccipital bone scores: 1, well ossified; 5, completely unossified. (B) The incidence of fetus with sternum malformations. (C) The incidence of fetus with rib malformations. Data were presented as means ± S.E.M. ^**^ *P*<0.01, ^*^ *P*<0.05.

The effects of melatonin on LPS-induced skeletal malformation were then analyzed. As shown in Figure 2A, a five-day LPS injection obviously retarded fetal supraoccipital ossification. Of interest, orally administered melatonin significantly alleviated LPS-induced supraoccipital ossification retardation. The effects of melatonin on LPS-induced sternum malformation were analyzed. As shown in Figure 2B, 62.37% of fetuses were with sternum malformation in the LPS group. The incidence of fetuses with sternum malformation declined to 32.30% in dams orally administered with melatonin before LPS injection. The effects of melatonin on LPS-induced rib malformation are presented in Figure 2C. As expected, 67.68% of fetuses were with rib malformation in the LPS group. The incidence of fetuses with rib malformation declined to 24.69% in dams orally administered with melatonin before LPS injection.

### Melatonin attenuates LPS-induced disturbance of folate transport

**Figure 3:**
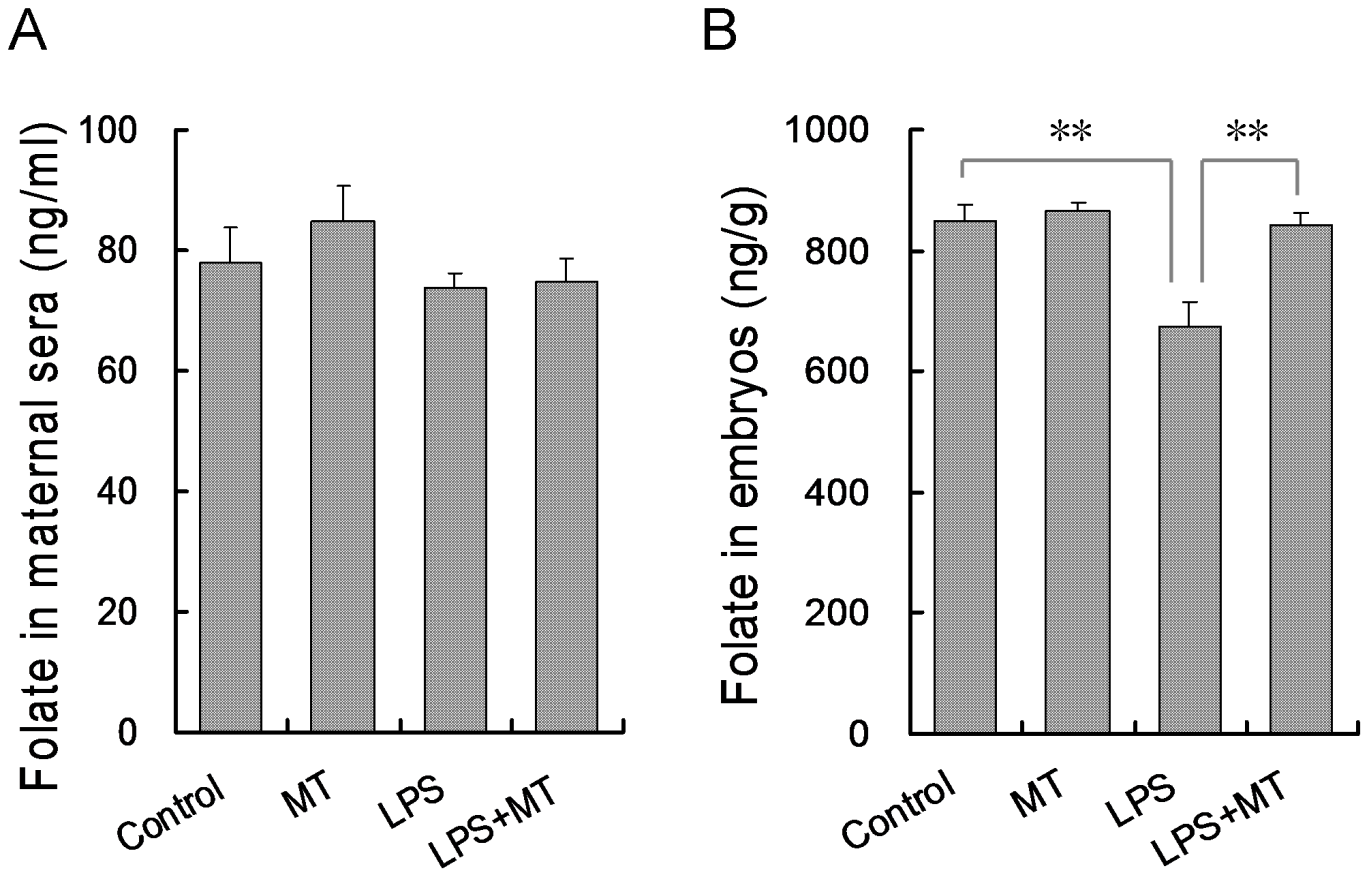
Effects of melatonin on folate contents in maternal sera and embryos. In LPS group, pregnant mice were i.p. injected with LPS (25 µg/kg) daily from GD8 to GD12. In LPS+melatonin (MT) group, pregnant mice were orally administered with melatonin (50 mg/kg) 1 h before each LPS injection. In control group, pregnant mice were i.p. injected with NS daily from GD8 to GD12. In melatonin alone group, pregnant mice were orally administered with melatonin (50 mg/kg) 1 h before each NS injection. All pregnant mice were sacrificed 12 h after the last LPS injection. Folate contents in maternal sera and embryos were measured. Data were presented as means ± S.E.M. of twelve samples from twelve different pregnant mice. ^**^ *P*<0.01, ^*^ *P*<0.05.

The effects of maternal LPS exposure on folate content in maternal sera and embryos were analyzed. As shown in Figure 3A, a five-day LPS injection had little effect on folate concentration in maternal sera. Of interest, embryonic folate content was markedly reduced in LPS-treated mice as compared with controls (Figure 3B). The effects of melatonin on LPS-induced reduction of embryonic folate content were then evaluated. Although melatonin alone had no effect on embryonic folate content, orally administered melatonin alleviated LPS-induced reduction of embryonic folate content (Figure 3B).

### Melatonin attenuates LPS-induced down-regulation of folate transporters

**Figure 4:**
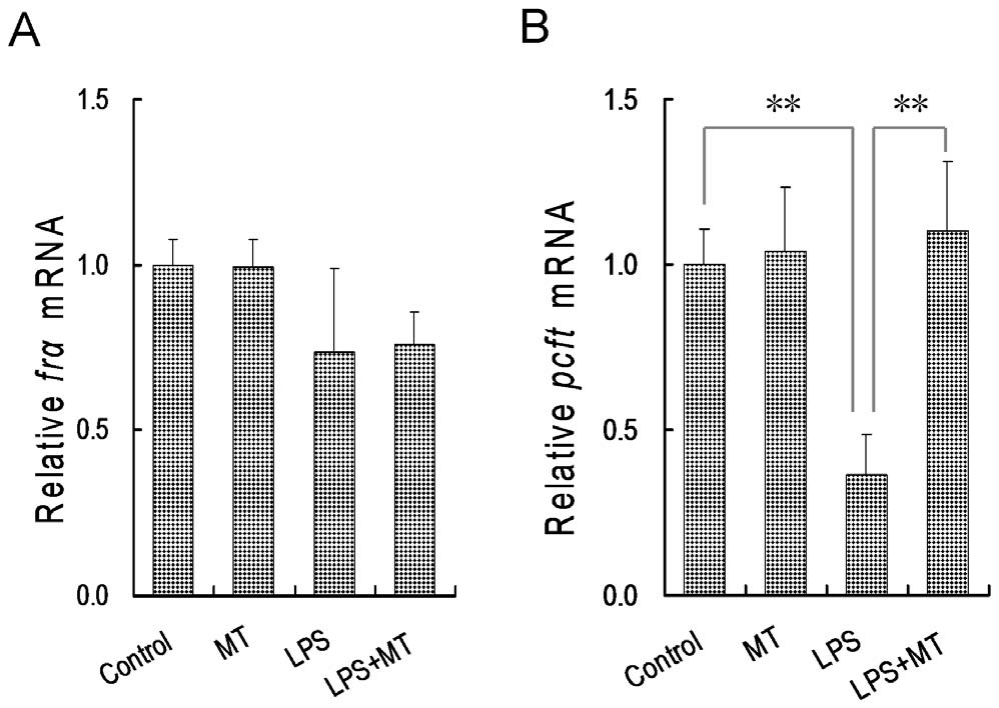
Effects of melatonin on the expression of frα and pcft in mouse placentas. In LPS group, pregnant mice were i.p. injected with LPS (25 µg/kg) on GD10. In LPS+melatonin (MT) group, pregnant mice were orally administered with melatonin (50 mg/kg) 1 h before LPS injection. In control group, pregnant mice were i.p. injected with NS on GD10. In melatonin alone group, pregnant mice were orally administered with melatonin (50 mg/kg) 1 h before NS injection. All pregnant mice were sacrificed 12 h after LPS injection. Placental frα and pcft mRNAs were measured using real-time RT-PCR. (A) frα; (B) pcft. Data were presented as means ± S.E.M. of six samples from six different pregnant mice. ^**^ *P*<0.01, ^*^ *P*<0.05.

The effects of melatonin on the expression of placental *folate receptor alpha* (*fr*α) were evaluated. As shown in Figure 4A, there was no significant difference on the expression of placental *fr*α among different groups. The effects of melatonin on the expression of placental folate transporters were then analyzed. As shown in Figure 4B, mRNA level of placental *proton-coupled folate transporter* (*pcft*) was significantly reduced 12 h after LPS injection. Although melatonin did not affect the expression of placental *pcft*, orally administered melatonin significantly inhibits LPS-induced down-regulation of *pcft* in mouse placenta.

### Melatonin attenuates LPS-induced placental inflammatory cytokines

**Figure 5:**
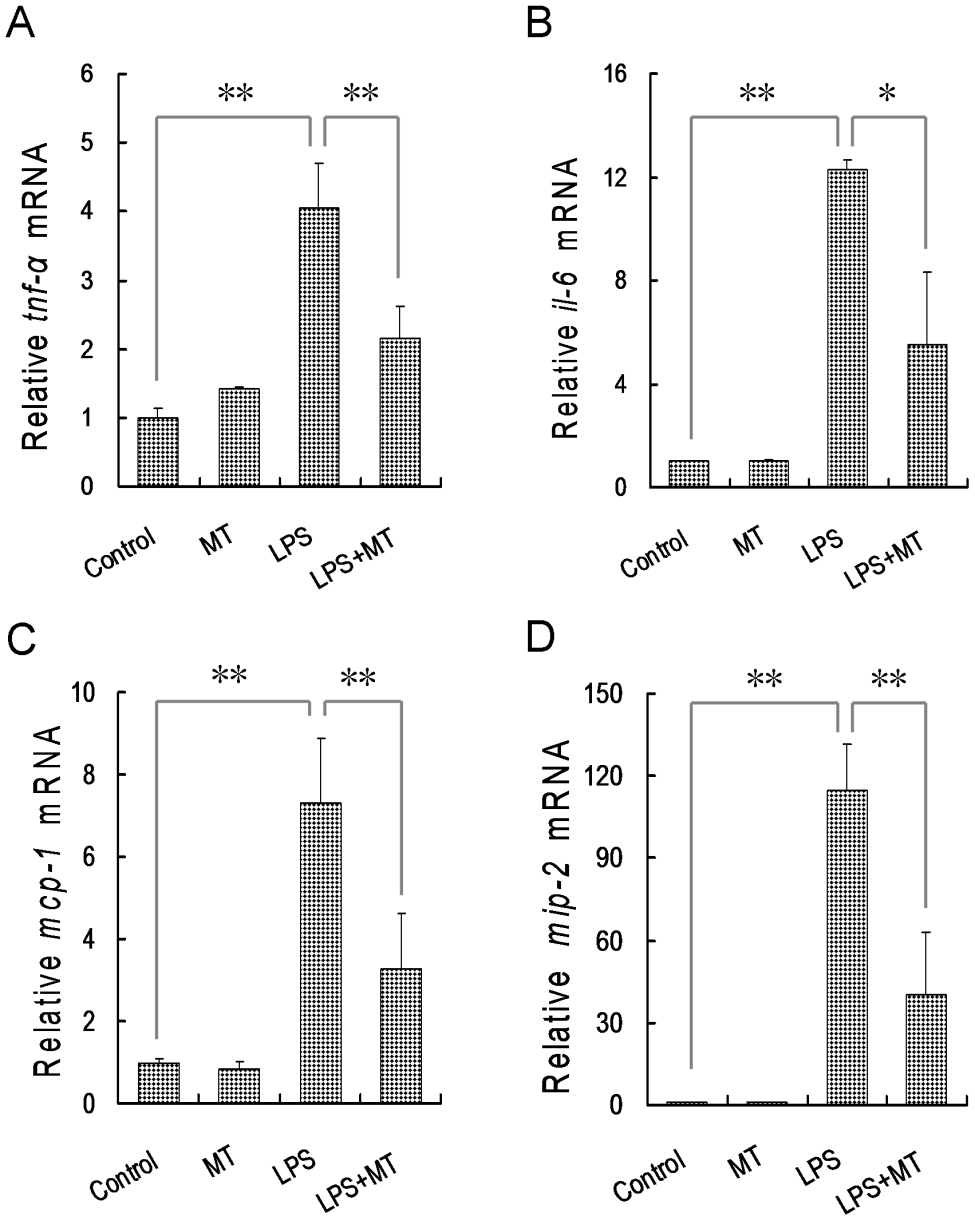
Effects of melatonin on LPS-induced placental inflammatory cytokines and chemokines. In LPS group, pregnant mice were i.p. injected with LPS (25 µg/kg) on GD10. In LPS+melatonin (MT) group, pregnant mice were orally administered with melatonin (50 mg/kg) 1 h before LPS injection. In control group, pregnant mice were i.p. injected with NS on GD10. In melatonin alone group, pregnant mice were orally administered with melatonin (50 mg/kg) 1 h before NS injection. All pregnant mice were sacrificed 2 h after LPS injection. Placental tnf-α, il-6, mcp-1 and mip-2 mRNAs were measured using real-time RT-PCR. (A) tnf-α; (B) il-6; (C) mcp-1; (D) mip-2. All data were expressed as means ± S.E.M of six samples from six different pregnant mice. ^**^ *P*<0.01, ^*^ *P*<0.05.

The effects of melatonin on LPS-induced placental inflammatory cytokines were analyzed. As shown in Figure 5A and 5B, the expressions of placental *tnf-*α and *il-6*, two inflammatory cytokines, were significantly increased 2 h after LPS injection. Of interest, orally administered melatonin almost completely repressed LPS-induced up-regulation of inflammatory cytokines in mouse placentas. The effects of melatonin on LPS-induced placental inflammatory cytokines are presented in Figure 5C and 5D. As expected, the expressions of placental *mcp-1* and *mip-2*, two chemokines, were significantly increased 2 h after LPS injection. Orally administered melatonin markedly inhibited LPS-induced up-regulation of chemokines in mouse placentas.

## Discussion

According to an earlier report from our laboratory, injection with melatonin ameliorated LPS-induced fetal demise and IUGR [24]. A recent study showed that subcutaneous implantation with melatonin protected against LPS-induced preterm delivery [25]. The present study investigated the effect of orally administered melatonin on LPS-induced NTDs. Our results showed that orally administered melatonin reduced the incidence of LPS-induced NTDs from 27.5% to 13.3%. Moreover, orally administered melatonin significantly protected against LPS-induced skeletal malformation. These results suggest that melatonin prevents not only LPS-induced preterm delivery and IUGR but also NTDs and skeletal malformation, which expands the protective effects of melatonin against LPS-induced developmental toxicity.

Folate plays a crucial role on one-carbon metabolism for physiological DNA synthesis and cell division [27]. Increasing evidence demonstrates that maternal folate deficiency during pregnancy is major etiology for fetal NTDs [28]. Indeed, a recent report from our laboratory showed that maternal folate supplementation during pregnancy prevented LPS-induced NTDs in mice [9]. Thus, it is especially interesting whether maternal LPS exposure influences folate metabolism. To test this hypothesis, we measured folate content in maternal sera and embryos. Despite of no difference on folate content in maternal sera between LPS-treated dams and controls, embryonic folate content was markedly reduced when pregnant mice were injected with LPS during organogenesis, indicating that maternal LPS exposure disturbs folate transport from maternal circulation into the fetus. Interestingly, LPS-induced disturbance of folate transport was attenuated when pregnant mice were orally administered with melatonin before LPS injection. Indeed, the placentas mediate uptake of folates from maternal circulation to the developing embryos, which are critically important for cell proliferation and the development of critical structures, such as the embryonic neural tube [29]. Taken together, these results suggest that melatonin protects against LPS-induced NTDs, at least partially, through attenuating LPS-induced disturbance of folate transport from maternal circulation through the placenta into the fetus.

The transport of folate from maternal circulation through the placenta into the fetus is mediated by several specific transporters, including folate receptors (mainly FRα) and folate transporters (mainly PCFT) [30]. According to several recent reports, FR-α and PCFT are highly expressed in human and rodent placentas during early embryonic development [31], [32]. The present study showed that no significant difference on the expression of placental *fr*α was observed among different groups. By contrast, *pcft*, major folate transporter in placenta, was markedly down-regulated when pregnant mice were injected with LPS during organogenesis. As melatonin alleviates LPS-induced disturbance of folate transport from maternal circulation through the placenta into the fetus, we further analyze the effects of melatonin on placental folate transporters. As expected, melatonin alone had no effect on the expression of folate transporters in placentas. Interestingly, LPS-induced down-regulation of placental *pcft* was obviously attenuated in mice pretreated with melatonin. These results suggest that melatonin improves folate transport from maternal circulation into the fetus through attenuating LPS-induced down-regulation of *pcft*.in placenta.

The mechanism through which melatonin attenuates LPS-induced down-regulation of placental *pcft* remains obscure. Increasing evidence demonstrates that melatonin has an anti-inflammatory effect [33]–[36]. According to an earlier report from our laboratory, orally administered melatonin during pregnancy inhibits LPS-evoked release of TNF-α in maternal serum [20]. The present study found that orally administered melatonin during pregnancy almost completely repressed LPS-induced up-regulation of *tnf-*α, *il-6*, *mcp-1* and *mip-2* in mouse placenta. Although no direct evidence demonstrated that inflammatory cytokines could regulate placental folate transporters, TNF-α, a major inflammatory cytokine, significantly repressed folate uptake by human placental BeWo cells [37], indicating that TNF-α is, at least partially, involved in LPS-induced down-regulation of placental *pcft*. Therefore, we speculate that melatonin might attenuate LPS-induced down-regulation of placental *pcft* through its anti-inflammatory activity.

According to an earlier report, maternally administered melatonin prevented ischemia/reperfusion-induced placental oxidative mitochondrial damage and IUGR [38]. Another study found that orally administered melatonin significantly improved placental efficiency and birth weight by upregulating placental antioxidant enzymes [39]. A recent report showed that antenatal melatonin administration significantly reduced fetoplacental oxidative stress and prevented IUGR-associated newborn neurodevelopmental deficits and brain injury [40]. The present study found that orally administered melatonin protected against LPS-induced NTDs through attenuating LPS-induced disturbance of folate transport from maternal circulation through the placenta into the fetus. However, the present study has several limitations. First, the present study only investigated the effects of a single dose melatonin on LPS-induced placental folate transport and fetal NTDs. Second, the present study only investigated the effects of pretreatment with melatonin on LPS-induced placental folate transport and fetal NTDs. Thus, additional work is required to determine a dose response curve to assess the effects of different doses melatonin on LPS-induced placental folate transport and fetal NTDs. In addition, the protective effects of melatonin against NTDs need to be demonstrated in different models of NTDs.

In summary, the present study demonstrates for the first time that orally administered melatonin attenuates LPS-induced down-regulation of placental folate transporters. Correspondingly, orally administered melatonin alleviates LPS-induced disturbance of folate transport from maternal circulation into the fetus through its anti-inflammatory activity. Importantly, orally administered melatonin prevents LPS-induced NTDs. Thus, melatonin may have a potential preventive utility for protecting against inflammation-associated fetal NTDs.
